# Surgical management of vascular anomalies in children at a tertiary care hospital in a resource-limited setting: a Tanzanian experience with 134 patients

**DOI:** 10.1186/s13104-015-1718-3

**Published:** 2015-11-30

**Authors:** Phillipo L. Chalya, Neema M. Kayange, Peter F. Rambau, Mange Manyama, Japhet M. Gilyoma

**Affiliations:** Department of Surgery, Bugando Medical Centre, Mwanza, Tanzania; Department of Paediatrics, Bugando Medical Centre, Mwanza, Tanzania; Department of Pathology, Catholic University of Health and Allied Sciences-Bugando, Mwanza, Tanzania; Department of Anatomy, Catholic University of Health and Allied Sciences-Bugando, Mwanza, Tanzania; Department of Otorhinolaryngology, Catholic University of Health and Allied Sciences-Bugando, Mwanza, Tanzania

**Keywords:** Vascular anomalies, Children, Surgical management, Challenges, Outcome, Tanzania

## Abstract

**Background:**

Vascular anomalies pose major diagnostic and therapeutic challenges among pediatricians and pediatric surgeons practicing in resource limited countries. There is paucity of published data regarding this subject in Tanzania and Bugando Medical Centre in particular. This study describes our experiences on the challenges and outcome of surgical management of childhood vascular anomalies in our environment.

**Methods:**

Between January 2009 and December 2013, a prospective study on the surgical management of vascular anomalies was undertaken at Bugando Medical Centre.

**Results:**

A total of 134 patients (M; F = 1:2.5) were studied. The median age at presentation was 6 years. Of the 134 patients, 101 (75.4 %) were diagnosed as having vascular tumors and 33 (24.6 %) had vascular malformations. The head and the neck were the most frequent anatomical site recorded as having a tumor (56.7 % of patients). Out of 134 patients, 129 (96.3 %) underwent surgical treatment. Failure to respond to non-operative treatment (86.8 %), huge disfiguring/obstructing mass (4.7 %), infection (3.1 %), ulceration (3.1 %) and hemorrhage (2.3 %) were indications for surgical intervention. Tumor excision and primary wound closure was the most common type of surgical procedure performed in 80.6 % of patients. Surgical site infection was the most frequent complications accounting for 33.8 % of cases. Mortality rate was 1.5 %. Tumor excision and primary wound closure gave better outcome compared with other surgical options (p < 0.001). Outcome of injection sclerotherapy in 3 (3.7 %) children, serial ligation of feeder vessels employed in 2 (1.6 %), and conservative treatment in 5 (3.7 %), were poor and required conversion to surgical excision. Despite low mortality rate recorded in this study, but ugly scar, 14 (20.6 %) and limb deformity, 6 (8.8 %) were problems. The overall result of surgical treatment at the end of follow up period was excellent in 108 (87.1 %) patients.

**Conclusion:**

Surgical excision and primary wound closure gave good outcome which could be employed in complicated and vascular anomalies which failed to respond to other treatment in regions with limited resources.

## Background

Vascular anomalies, defined as congenital lesions of abnormal vascular development, encompass a wide spectrum of lesions with varying degrees of severity, ranging from isolated and innocuous lesions, to those that are disfiguring and disabling, to those that signal the presence of a more complex syndrome [1–9].

The classification system, originally proposed by Mulliken and Glowacki [10] and later updated by the International Society for the Study of Vascular Anomalies [11] divides vascular anomalies into vascular tumours and vascular malformations, based on their endothelial cell characteristics, clinical presentation, natural history, and histopathological characteristics.

Both vascular tumors and malformations may occur anywhere on the body. Hemangiomas are the most common vascular tumors in children that are rarely apparent at birth, grow rapidly during the first 6 months of life, involute with time and do not necessarily infiltrate but can sometimes be destructive [1–3]. They occur with an incidence of 1–3 % in newborns and this increase up to 10–12 % by the end of 1 year [4]. They are more frequent in females than in males, with a ratio of 3:1 [5]. They appear even more frequently among premature babies [4, 5]. These tumors may be syndromic or non-syndromic and are rarely present at birth but appear during the second to third weeks of life as bluish, pink or reddish subcutaneous lesions of any shape that may rapidly increase in size [6, 7]. The lesion is composed of immature vasoformative tissue that often involves the subcutaneous tissue as well as the skin and in severe cases the muscles may also be involved [7]. Although the lesions are benign and composed of vascular tissues that are haphazardly arranged, life threatening complications such as bleeding, ulceration and infection have been reported in many series [10–14].

The treatment of vascular anomalies is a new and rapidly developing discipline that demands teamwork between the pediatricians, plastic surgeon, interventional radiologist, paediatric surgeon, otorhinolaryngologist and paediatric oncologist [11–14]. Various modality of treatment which includes conservative treatment, surgical excision, injection sclerotherapy, cryotherapy, laser treatment, angiographic embolization, angioplasty/binding of feeder vessels, use of angiogenesis inhibitor drugs, and especially the use of corticosteroids which are readily available have given good results in many centers [6, 12, 15, 16]. However, in many evolving pediatric surgical centers particularly in sub-Saharan Africa, there are limited approaches to management owing to lack of facilities [17]. The management of vascular anomalies in such resource-limited centers remains a major challenge especially when the lesion is located in sensitive places and in patients presenting with life threatening complications [16, 17]. There are, however, paucity of published local data regarding the challenges and outcome of surgical management of childhood vascular anomalies in this Africa sub-region [17]. The aim of this study was to describe our own experience on the surgical management of vascular anomalies in children outlining the role of surgery in the management of very symptomatic vascular anomalies that may not respond to other modalities of treatment.

## Methods

### Study design and setting

This was a descriptive prospective study on the surgical management of childhood vascular anomalies which was conducted at Bugando Medical Centre over a period of 5 years between January 2009 and December 2013. Bugando Medical Centre is the only tertiary health institution serving the whole of the northwestern part of Tanzania, serving a population of about 13 millions. It is a 1000 bed referral hospital located in Mwanza city in the northwestern Tanzania on the southern border of Lake Victoria. It is also a teaching hospital for the Catholic University of Health and Allied Sciences—Bugando (CUHAS—Bugando).

### Study population

The study included all the children who were referred from pediatric department of Bugando Medical Centre and other hospitals in neighboring regions after failure of at least 5 years of non-operative management and/or complicated lesions were included in the study. Those whose lesions resolved spontaneously and/or during non operative treatment follow-up were excluded from the study. The diagnosis of vascular anomalies was made based on clinical and imaging characteristics of the lesion and confirmed histopathologically. Imaging investigations were ultrasound and Doppler ultrasound. Routine investigations included hemoglobin estimation, full blood picture, liver function tests, bleeding indices, renal function tests etc’.

In view of the lack of adequate facilities required for injection sclerotherapy, cryotherapy, laser treatment, angiographic embolization, angioplasty/binding of feeder vessels, and use of angiogenesis inhibitor drugs, the majority of patients underwent tumour excision with either primary closure or delayed closure.

Data were collected using a pre-formed questionnaire. Included in the questionnaire were age of the patient, sex, associated malformations, anatomical site, type and size of the lesion, radiological findings, treatment offered, treatment outcome and follow up of patients.

### Statistical data analysis

The statistical data analysis was performed using statistical package for social sciences (SPSS) version 17.0 for Windows (SPSS, Chicago, IL, USA). The median + interquartile range (IQR) and ranges were calculated for continuous variables whereas proportions and frequency tables were used to summarize categorical variables. Chi square (χ^2^) test were used to test for the significance of association between the independent (predictor) and dependent (outcome) variables in the categorical variables. The level of significance was considered as p < 0.05. Study variable that was found to be statistically significant in univariate analysis were subjected to multivariate logistic regression analysis. Multivariate logistic regression analysis was used to determine predictor variables that predict the postoperative complications, hospital stay and mortality.

### Ethical consideration

Ethical approval to conduct the study was obtained from the CUHAS-Bugando/BMC joint institutional ethic review committee before the commencement of the study. An informed written consent was sought from the parents or guardians.

## Results

### Patient’s characteristics

During the period of study, a total of 148 patients with vascular anomalies were referred to the paediatric surgical unit for the surgical treatment of their lesions. Of these, 14 were excluded from the study due to failure to meet the inclusion criteria. Thus 134 patients were enrolled in the study. Their age at presentation ranged between a day and 10 years with a median of 6 years (IQR = 2–6 years). Out of 134, 96 (71.6 %) were females and the remaining 38 (28.4 %) were males with a female to male ratio of 2.5:1.

### Clinical presentation

Of the 134 patients, 101 (75.4 %) were diagnosed as having vascular tumors [i.e. 98 (97.0 %) hemangiomas and 3 (3.0 %) Kaposiform hemangioendothelioma] and 33 (24.6 %) had vascular malformations. The lesions were present at birth in 44 (32.8 %) babies while it appeared between 2 and 3 weeks in 80 (59.7 %), and after 6 months in 10 (7.5 %). The majority of patients, 120 (89.6 %) were symptomatic presenting with a local swelling or mass, with or without associated skin discoloration, 14 (10.4 %) patients were asymptomatic and discovered incidentally (skin discoloration and/or mild swelling) during a health check.

Out of 134 patients, 128 (95.5 %) were non-syndromic and 6 (4.5 %) patients were syndromic having other associated malformations such as congenital hernia in two patients and club foot and undescended testis in one patient each, respectively. The head and the neck were the most frequent anatomical site recorded as having a tumor (56.7 % of patients) (Table 1). The size of lesions ranged from spot-like lesion to extensive lesion measuring about 12 × 15 cm in diameter, and from a flat lesion to a huge mass. One patient presented with acute abdomen due to ruptured hepatic hemangiomas and three (2.3 %) patients with Kaposiform hemangioendothelioma had associated thrombocytopenia and presented with huge tumors and recurrent bleeding.

**Table 1:** Distribution of patients according to anatomical site

Anatomical sites	Frequency	Percentages
Head and neck (including face, cheek, palate, orbit, maxilla, scalp)	93	56.7
Lower limbs (gluteus, thigh, calf)	32	19.5
Upper limbs (including shoulder, scapula, arm, forearm, hand)	24	14.6
Trunk (abdomen and chest wall)	11	6.7
Axilla	3	1.8
Liver	1	0.6

### Radiological findings

Abdominal ultrasound revealed hepatic hemangioma in 1 patient. On ultrasound, hemangiomas manifested as mixed echogenicity but predominantly hypoechoic. Doppler ultrasound performed in 56 patients demonstrated vascular anomalies in 52 patients. MRI was not performed due to lack of this facility at our centre.

### Treatment modalities

Out of 134 patients, 129 (96.3 %) underwent surgical treatment and the remaining five (3.7 %) patients were treated conservatively by non-surgical approach (observation alone). Tumor excision and primary was the most common surgical procedure performed accounting for 80.6 % of patients (Table 2). In this study, multiple excisions were required to completely eradicate the lesion in 15 (11.2 %) patients.

**Table 2:** Distribution of patients according to surgical procedure performed (N = 129)

Surgical procedure performed	Frequency	Percentages
Tumor excision/primary closure	104	80.6
Tumor excision/delayed skin graft	8	6.2
Tumor excision/delayed closure	5	3.9
Tumor excision/immediate flap	3	2.3
Tumor excision/immediate skin graft	3	2.3
Sclerotherapy (5 % phenol in oil)	3	2.3
Serial feeding vessels ligation	2	1.6
Exploratory laparotomy/(inoperable tumor)	1	0.8

### Treatment outcome and follow up of patients

Postoperative complications were recorded in 68 patients giving a complication rate of 50.7 %. Out of the 68 complications, surgical site infection was the most frequent complications accounting for 33.8 % of cases (Table 3). All postoperative complications were treated conservatively except graft failure in two patients and ugly scar in six patients which required surgical correction. Tumor excision and primary wound closure gave better outcome compared with other surgical options (p < 0.001). In this study, two patients died giving a mortality rate of 1.5 %. The causes of death in these two patients were intraoperative cardiac arrest and disseminated intravascular coagulopathy resulting from severe intraoperative bleeding due to spontaneous rupture of hepatic hemangioma. Outcome of injection sclerotherapy in 3 (3.7 %) children, serial ligation of feeder vessels employed in 2 (1.6 %), and conservative treatment in 5 (3.7 %), were poor and required conversion to surgical excision. The overall result of surgical treatment at the end of follow up period was excellent in 108 (87.1 %) patients.

**Table 3:** Distribution of patients according to postoperative complications (N = 68)

Postoperative complications	Frequency	Percentages
Surgical site infection	23	33.8
Poor cosmetic results	21	30.9
Ugly scar	14	20.6
Limb deformity	6	8.8
Graft failure	2	2.9
Others	2	2.9

## Discussion

Vascular anomalies pose major diagnostic and therapeutic challenges among pediatricians and pediatric surgeons practicing in resource limited countries [17]. In this study, the median age of patients at presentation was 6 years which is comparable with that reported by Osifo and Evbuomwan [18] in Nigeria. This study showed that females were more affected than males with a female to male ratio of 2.5:1. This agrees with other studies which reported female predominance [19, 20], but at variance with Osifo and Evbuomwan [18] in Nigeria who reported that males were more affected than females. The reason for this gender differences is unclear and warrants further investigation.

In the present study, the lesions were present at birth in 44 (32.8 %) babies while it appeared between 2 and 3 weeks in 80 (59.7 %), and after 6 months in 10 (7.5 %). This observation concurs with one study in Nigeria [18, 21]. Rarely, hemangiomas can be present as a fully grown lesion at birth, and has also been reported to be diagnosed by antenatal ultrasound and these lesions which are now termed ‘Congenital Haemangiomas’ are histologically distinct from haemangioma of infancy. They generally do not require surgical intervention [22]. Most infantile hemangiomas are not present at birth, so may be many of these lesions in our study were really vascular malformations and not really infantile hemangiomas. This misclassification may be a result of lack of advanced immunohistochemical stains such as immunostain Glut1 leading to difficulty in categorizing hemangiomas and vascular malformation into separate entities.

In agreement with other studies [18, 20], the majority of patients in this study were symptomatic at the time of presentation. This can be explained by the fact that the majority of patients in this study were referred from pediatric department of this hospital and other hospitals in neighboring regions after failure of non operative management or as a result of life threatening complications and these patients presented with symptomatic lesions.

Although vascular anomalies can occur anywhere, the head and the neck is the site of predilection [23]. Finn et al. [24] in a large series, found that 60 % of vascular anomalies occurred on the head and the neck, 25 % on the trunk, and 15 % on the extremities. This clinical finding agrees with our study in which head and the neck was the most frequent anatomical site recorded in 56.7 % of patients; but at variance with Osifo and Evbuomwan [18] in Nigeria who reported upper limbs as the most common anatomical site recorded. We could not find in literature the reasons for this anatomical site differences.

In the majority of vascular anomalies, a clinical diagnosis can be accurately made based on history of timing and physical characteristics of the lesions (such as size, color, surface characteristics, and tactile qualities) [20, 21]. However, differentiating between a deep hemangiomas and a vascular malformation may be difficult. The advent of new immunohistochemical stains such as immunostain Glut1 is finally bringing pathological practice into line with the clinical classification. Doppler ultrasound is the least invasive and the most cost-effective imaging modality for hemangiomas and other vascular anomalies. It is recommended as the initial test of choice to differentiate between vascular tumors and malformations [25]. In the current study, Doppler ultrasound performed in only 56 patients demonstrated hemangiomas/vascular malformations in 52 patients. Abdominal ultrasound was used to detect hepatic hemangioma in one patient who presented with right upper abdominal pain and swelling.

Surgical excision was the only available and affordable treatment option for the referred children owing to lack of modern facilities that are currently in use in other centers [6, 15, 16, 26]. Utilization of such facilities would have lessened surgical burden and the postoperative deformity and poor cosmetic results in lesions which involved the limbs and face/neck. Nevertheless, lesions which were amenable to surgical excision and primary wound closure gave overall best cosmetic results followed by excision and primary skin graft.

In this study, failure to respond to non-surgical treatment, presence of huge disfiguring or obstructing mass and associated life-threatening complications such as hemorrhage, ulceration and infection were the main indications which necessitated surgical intervention as recorded in other studies [18, 27]. Propranolol is a cheap and highly effective treatment for hemangiomas of infancy, with a response rate upwards of 90 % [5]. It is therefore a good treatment for use in developing countries like Tanzania. In this study propranolol and steroids were used in some patients with good results and only patients who failed to respond to these drugs were referred for surgery. Failure to respond to propranolol/steroids may be explained by the fact that the first use of these drugs were in large, disfiguring ‘hemangiomas’ which may be venous malformations, where the drug has no efficacy. In developing countries where new immunohistochemical stains such as immunostain Glut1 has limited availability, it is common to see vascular malformations which have ‘failed’ inappropriate treatment with steroids and/or propranolol, a practice which can be traced back to the continued inappropriate use of the term ‘hemangioma’ by pathologists. Associated life-threatening complications such as hemorrhage, ulceration and infection influenced the choice of delayed surgical wound closure with poor cosmetic results. Infected massive vascular lesions required incision and drainage, dressing and use of broad spectrum antibiotics before excision. This increased the duration of hospitalization and cost of treatment compared to children with similar uninfected lesions.

The use of injection sclerotherapy and selective feeder vessels ligation were associated with complications and poor results which necessitated conversion to surgical excision. This could be because 5 % phenol in almond oil was the only available and affordable sclerosant in the center. Other authors recorded good successes with the use of hypertonic saline as sclerosant in their centers which was not attempted in this study [7, 12]. The lack of facilities required for angiography ligation and embolization made outcome of selective ligation of feeder vessels very poor because identification of the major feeding vessels was difficult and the occlusion of the major draining vessels required to retain the sclerosant to the lesion was not possible. In any case, ligation of feeding vessels is recognized not to be an effective long term treatment for vascular malformations or hemangiomas.

Facial involvement posed cosmetic challenge but non availability of other treatment options and the failure of spontaneous involution and injection sclerotherapy influenced the choice of surgical excision as a last resort. However, excision with primary wound closure and/or primary skin graft gave the best results in lesions located in this region among the surgical options.

As reported in other studies, all hemangiomas were said to involute during childhood [7, 28]. Many hemangiomas that involuted spontaneously did not seek surgical consultation in this setting. Lips involvement, though less commonly encountered, posed a unique challenge as complete single stage excision of the majority of hemangiomas/vascular anomalies in this location was not possible. The rapid recurrence and incomplete excision recorded required multiple secessions to completely eradicate the tumor as also reported by others [29]. In this study, multiple excisions were required to completely eradicate the lesions in 15 (11.2 %) patients.

The three children with thrombocytopenia who presented with massive tumor and recurrent bleeding posed a major challenge in this study unlike reports from more equipped settings [7, 14, 30, 31]. Multiple transfusions of whole blood and platelet concentrate followed by excision and myocutaneous flap were able to save the lives of affected children though some healed with residual deformity.

Rupture of hepatic hemangioma with hemoperitonium, is the most dreaded complication and often fatal if not promptly managed [32]. Intraperitoneal bleeding from ruptured hepatic hemangioma, which can either occur spontaneously or due to trauma, is a rare life- threatening complication [33]; it occurs in about 4 % of patients but is fatal in 60–75 % [34]. In a recent review of literature, the operative mortality rate of ruptured hemangioma was reported to be 36.4 % [32]. In the current study, one patient who had hepatic hemangioma presented with acute abdomen shock and hemoperitonium due to ruptured giant hepatic hemangioma underwent emergency laparotomy. However, the patient died intraoperatively due to disseminated intravascular coagulopathy.

The major limitation of this study, however, was the difficulty in categorizing hemangiomas and vascular malformation into separate entities as have been emphasized by other authors [1, 2, 7, 18] who stressed this as the major factor determining the choice of management. In this study, lack of new immunohistochemical stains such as immunostain Glut1 and advanced imaging such as MRI made the classification of lesions with atypical presentation difficult and this might have led to misclassification of lesions. In centers with sophisticated facilities, surgical excision would have been avoided in many cases which make findings from this study applicable mainly to centers with limited resources.

## Conclusion

Vascular anomalies are the most common tumors in infancy and childhood and pose major diagnostic and therapeutic challenges in our environment. Making the correct clinical diagnosis is of critical importance. In particular it is important to correctly identify hemangiomas of infancy which can be treated with propranolol in a resource poor environment. Surgical management of vascular anomalies was challenging owing to involvement of sensitive places, huge lesions involving all soft tissues and presentation with life threatening complications. Excision with primary wound closure and primary skin grafts gave overall best outcome in this setting. Complicated lesions and those which failed to respond to non operative treatment should be offered surgical excision and primary wound closure when possible in regions with limited resources. For vascular malformations, which are a more difficult problem, although interventional radiology treatments are widely described, in resource poor settings where radiological equipment and expertise is not available, surgical management should still be offered and can provide satisfactory outcomes.
